# Integrating SMS Text Messages Into a Preventive Intervention for Postpartum Depression Delivered via In-Home Visitation Programs: Feasibility and Acceptability Study

**DOI:** 10.2196/30995

**Published:** 2021-11-18

**Authors:** Alinne Z Barrera, Jaime Hamil, Darius Tandon

**Affiliations:** 1 Department of Psychology Palo Alto University Palo Alto, CA United States; 2 Institute for Public Health and Medicine Center for Community Health Northwestern University Feinberg School of Medicine Chicago, IL United States

**Keywords:** perinatal mental health, postpartum depression, public health, SMS, technology

## Abstract

**Background:**

The Mothers and Babies (MB) Course is recognized by the US Preventive Services Task Force as an evidence-based preventive intervention for postpartum depression (PPD) that should be recommended to pregnant women at risk for PPD.

**Objective:**

This report examines the feasibility and acceptability of enhancing the MB 1-on-1 intervention by adding 36 SMS text messages that target 3 areas: reinforcement of skills, between-session homework reminders, and responding to self-monitoring texts (ie, MB Plus Text Messaging [MB-TXT]).

**Methods:**

In partnership with 9 home visiting programs, 28 ethnically and racially diverse pregnant women (mean 25.6, SD 9.0 weeks) received MB-TXT. Feasibility was defined by home visitors’ adherence to logging into the HealthySMS platform to enter session data and trigger SMS text messages within 7 days of the in-person session. The acceptability of MB-TXT was measured by participants’ usefulness and understanding ratings of the SMS text messages and responses to the self-monitoring SMS text messages.

**Results:**

On average, home visitors followed the study protocol and entered session-specific data between 5.50 and 61.17 days following the MB 1-on-1 sessions. A high proportion of participants responded to self-monitoring texts (25/28, 89%) and rated the text message content as very useful and understandable.

**Conclusions:**

This report contributes to a growing body of research focusing on digital adaptations of the MB course. SMS is a low-cost, accessible digital tool that can be integrated into existing interventions. With appropriate resources to support staff, it can be implemented in community-based organizations and health care systems that serve women at risk for PPD.

**Trial Registration:**

ClinicalTrials.gov NCT03420755; https://clinicaltrials.gov/ct2/show/NCT03420755

## Introduction

### Background

Women are at an increased risk of psychological distress during the perinatal period (ie, pregnancy and the first year post partum), with low-income women disproportionately affected. Regardless of the ethnic and racial background, low-income women report a 20% prevalence of postpartum depression (PPD) [1,2]. PPD is often preceded by symptoms of depression that are not sufficiently elevated to meet the criteria for a diagnosable disorder, but they negatively impact women and raise their risk for perinatal mood and affective disorders. When considering subthreshold depressive symptoms, the rate among low-income women is as high as 30% to 45% [3], regardless of factors that have been demonstrated to impact depression, such as race and ethnicity [4]. Unfortunately, perinatal depression is often undetected and untreated [5] despite mandates by professional organizations to regularly screen perinatal women for depression [6] and evidence to support the effective prevention and treatment of these disorders [7].

Multiple factors contribute to the disparity in psychological care received by perinatal women who suffer from depression. Providers, mainly obstetricians, often lack the time, knowledge, and training to appropriately identify women who meet the criteria for diagnosable depression or exhibit subthreshold symptoms [8,9]. It is hoped that this deficit in perinatal mental health care will improve given recent mandates expressed by the American College of Obstetricians and Gynecologists and the United States Preventive Services Task Force (USPSTF), with both organizations releasing strong statements recommending that women be regularly screened for depression during the perinatal period [6,10]. In 2019, the USPSTF took one step closer to improving the quality of mental health care for perinatal women when they provided evidence and support for the prevention of PPD interventions [11]. Of major significance was their statement that at-risk perinatal women exhibiting subthreshold symptoms or other risk factors of developing PPD should be referred to receive psychological interventions aimed at prevention of PPD and the subsequent negative consequences [12].

Perinatal women report barriers that interfere with their ability to advocate for their mental health needs, often expressing a lack of knowledge regarding psychological symptoms or help-seeking resources [13], as well as mental illness stigma or not fulfilling the role of motherhood [14,15]. Lack of transportation or childcare is a common barrier among women from low-income and ethnically diverse backgrounds [16,17]. Barriers to mental health care can be addressed through system-wide changes to how maternal mental health services are designed and implemented [17], such as through home visiting (HV) services where trained individuals meet regularly in-home, to assess family needs and provide services or referrals related to maternal and child health, parenting practices, child development, and school readiness. Similarly, there is a growing emphasis on integrating digital aspects into mental health services and interventions with growing evidence for their effectiveness among the general population (eg, Andrews et al, [18]). In fact, standalone and adjunct SMS programs for physical health are effective in promoting behavior change [19] and improving disease management, treatment compliance, and appointment attendance [20].

However, few digital tools have been adapted and tested to address the needs of perinatal women. This is surprising given the ubiquity of mobile devices and growing interest among perinatal women to access maternal health information on their digital devices [21,22]. Recent reports have supported the acceptability of programs and interventions for PPD that are web- or computer-based (eg, Ashford et al and Lau et al [23,24]), use short messaging service (SMS or text messaging) [25,26], and are designed within mobile apps; Hughson et al [27]. Most published digital programs for PPD are in the protocol, feasibility, or acceptability phases of development [28]. Regardless, in all instances, few low-income and culturally diverse women have taken part in these studies [29].

This study builds on a body of work focused on the implementation and testing of the Mothers and Babies (MB) Course, a PPD preventive intervention that is based on cognitive-behavioral therapy and social learning principles [30]. MB was 1 of only 2 psychological interventions recommended by the USPSTF for prevention of PPD based on a systematic review of 50 empirical studies [12]. MB aims to teach pregnant women skills to cope with changes in their mood and build healthy relationships with supportive adults and their newborn babies. MB has been examined in several controlled prevention trials, including as a face-to-face group intervention with high-risk Latina pregnant women [30,31], as a group intervention to augment HV programs [32-34], and online as a fully automated intervention, Mothers and Babies Online Course (eMB), that included an international sample of English- and Spanish-speaking pregnant women [13]. In-person trials have demonstrated that MB is effective at preventing the onset of PPD and reducing depressive symptoms when compared with usual care. While eMB did not demonstrate a statistical effect in preventing PPD, pregnant women with elevated depressive symptoms reported better depression outcomes if they engaged in eMB relative to those randomized to the information-only condition [35].

Despite the positive outcomes and the unique benefits of the MB modalities, difficulties with engagement and retention occurred across the MB modalities. Time commitment, transportation, childcare, and technology access uniquely impacted the effectiveness of each MB modality (group, 1-on-1, and web-based). For instance, the eMB provided participants with flexibility to engage with the course at their leisure and eliminated the need for transportation and childcare. However, the automated design and advances in technology have resulted in outdated methods to access or engage with web-based interventions. To address this challenge, a recent adaptation of the MB to an SMS platform [36] is in line with its widespread use and reliance on mobile devices. Similarly, the development of MB 1-on-1 intervention delivered individually to a single client as part of a HV program [37] reduces participant burden related to time constraints and childcare needs associated with the group modality. However, the home visitors’ ability to ensure immediate and long-term retention of skills taught during the MB 1-on-1 as well as between-session practicing of skills taught remains difficult to ascertain.

### Objectives

To maximize the benefits of more personalized and accessible adaptations of the MB intervention, experts in the delivery of MB partnered to create and examine the combination of MB 1-on-1 with SMS enhancements (ie, MB Plus Text Messaging [MB-TXT]). A small quasi-experimental study was conducted in which women were randomized to either MB 1-on-1 or MB-TXT (ie, MB 1-on-1 plus text messaging). The purpose of this report is to determine the feasibility and acceptability of MB-TXT across HV programs. We anticipated that the addition of SMS text messages to the MB-1-on-1 intervention would be acceptable, as measured by participants’ understanding of and engagement with the MB-TXT. The home visitors’ adherence to the MB-TXT protocol was also measured and is described in this report as part of examining how feasible the SMS enhancements were. A description of the full trial, including the psychological outcomes, is presented elsewhere.

## Methods

### Setting

The research team partnered with 9 HV programs in the Midwest to recruit women at high risk of poor pregnancy and parenting outcomes. Participant recruitment was conducted between June 2017 and July 2018. Data from the MB-TXT arm of this study are presented in this report. The Northwestern University Institutional Review Board approved all study procedures (STU00203918).

### Participants

The MB-TXT participants included 28 pregnant women (mean 25.6, SD 9.0 weeks) between the ages of 17 and 31 years (mean 23.7, SD 4.3 years) who mostly self-identified as Black or African American (12/28, 43%) or Hispanic or Latinx (10/28, 36%), and English-speaking (23/28, 82%). Participants were mostly single (16/28, 57%) or living with a partner (8/28, 29%) and stated that their pregnancy was unplanned (22/28, 79%). Demographic information and professional background of the home visitors were not collected as part of this study. See Table 1 for additional participant information.

**Table 1:** Participant characteristics (N=28).

Demographic characteristics		Value
Age (years), mean (SD)		23.7 (4.3)
**Race or ethnicity, n (%)**		
	Black or African American	12 (43)
	Hispanic or Latinx	10 (36)
	White	4 (14)
	Biracial	1 (4)
	Prefer to not specify	1 (4)
**Marital status, n (%)**		
	Single	16 (57)
	Living with partner	8 (28)
	Engaged	2 (7)
	Married	2 (7)
**Education, highest level, n (%)**		
	Grades 1-8	1 (4)
	Grades 9-12 (no diploma)	8 (28)
	High-school diploma or GED^a^	7 (25)
	Some college (no degree)	11 (39)
	College degree	1 (4)
Unemployed, n (%)		15 (54)
**Annual household income (US $), n (%)**		
	0-24,999	22 (81)
	25,000-49,999	2 (7)
	50,000-74,999	3 (11)
**English language, n (%)**		
	Primarily spoken at home	23 (82)
**Pregnancy characteristics**		
	Weeks pregnant, mean (SD)	25.6 (9.0)
	Unplanned pregnancy, n (%)	22 (78)
	First childbirth, n (%)	13 (46)

^a^GED: General Educational Development test.

### Intervention

MB 1-on-1 comprises 12 in-person sessions based on the original group MB [30], each lasting 15 to 20 minutes and delivered by trained home visitors. MB 1-on-1 is divided into 3 modules that correspond to key cognitive-behavioral elements: pleasant activities, thoughts (cognitions), and contact with others (social support). Throughout the MB, mood management skills are integrated with psychoeducational activities that encourage participants to understand the influence of their mood and cognitive-behavioral therapy components. The content is tailored to the specific needs and issues related to the perinatal period.

The MB-TXT includes the delivery of MB 1-on-1 intervention along with an added set of 3 SMS text messages after each of the 12 sessions (see Table 2). SMS text messages were designed to be triggered (ie, sent) following the completion of an MB 1-on-1 session. AZB developed 36 SMS text messages based on an earlier SMS version of the MB [36] to complement the MB 1-on-1 content. The MB 1-on-1 SMS text messages for each intervention session included 1 message focused on each of the following 3 areas: (1) skill reinforcement (eg, *some thoughts just come to us, but we can make a conscious effort to think of positive thoughts*); (2) homework reminders (eg, *what pleasant activity will you try today?*), and (3) self-monitoring (eg, *on a scale of 1-9, how would you rate your mood?*).

**Table 2:** Mothers and Babies Plus Text Messaging (MB-TXT) examples classified by Mothers and Babies (MB) 1-on-1 session topics.

Topic and HV^a^ #			Skill reinforcement		Homework reminder		Self-monitoring
**Course introduction**							
	1	Everybody has stress. It affects how you feel and how you interact with your baby. Do something today to manage stress. It can be as small as counting to 10		PP^b^: Watch and make notes about the 15 min video on being your child’s first teacher [38]		How stressed do you feel when you think about becoming or being a mom? Reply 9 to 1 (with 9 being the most stressed and 1 the least stressed)	
	2	Inner reality: your thoughts. Outer reality: what you do, who you relate to, what is around you. Stop and notice your realities		PP: Remember to keep track of how you are feeling by circling your mood rating on worksheet 2.2 at the end of the day		Reply and let us know what your mood is today on a scale of 1-9 (with 9 being the best day ever, 5 being average, and 1 worst day ever)	
**Pleasant activities**							
	3	It’s hard to do something pleasant when you are stressed or feeling sad. One pleasant activity can lead to more. Pick one thing you will do to destress		PP: What do you enjoy doing by yourself, with others, and with your baby? Think about it or write it down on worksheet 3.2		Are you working on creating your list of pleasant activities you like to do? Reply Y or N	
	4	Pleasant activities can be low to no cost, brief, and things that are part of our daily routines		PP: Do not forget to do 1 pleasant activity this week. Fit it into your week by planning a day/time to do it using worksheet 4.3		Were you able to schedule or complete a pleasant activity? Reply Y/N (If Y, also text us what you did!)	
	5	Babies learn by playing. Doing pleasant activities with you enhances their development. Notice how and what other babies in your community like to play		PP: Remember to keep track of how you are feeling (Quick Mood Scale) and how many pleasant activities you do each day on worksheet 5.3		Reply and let us know your mood for today on a scale of 1 to 9 (with 9 being the best)	
**Thoughts**							
	6	People have helpful and harmful thoughts and both types affect how we feel. Make an effort to think helpful thoughts or imagine positive images		PP: What helpful or harmful thoughts have you had this week about being pregnant or becoming a mom? Write them down on worksheet 6.6		Text us a positive thought you have about becoming or being a mom	
	7	Talking back to harmful thoughts is one way to reduce them. Today, change one of your harmful thoughts into a more helpful thought		PP: Keep track of your mood (Quick Mood Scale). Count how many helpful/harmful thoughts you have this week. Decrease harmful thoughts with a new skill on worksheet 7.4		Have you noticed any harmful thoughts you have? Reply Y/N (In addition, tell us if you used a strategy to reduce it.)	
	8	Your thoughts can affect your future and your baby’s future. It is helpful to have thoughts about your future so you can act in ways to achieve your goals		PP: This week think about what you want your baby’s ideal future to look like. What can you begin to do now to prepare for it? Put your ideas on worksheet 8.3		Reply and let us know your mood for today on a scale of 1-9 (with 9 being the best)	
**Contact with others**							
	9	Being with others can affect how you feel. Many people feel sad or depressed when they do not have positive contacts or have negative contacts with others		PP: On worksheet 9.4 rate how you are feeling and the number of positive and negative contacts you have each day. Try to do this every day of this week		Tell us about one positive contact you had this week. Text us who you were with and what you did together?	
	10	Support from others is important for all of us. Positive contacts help you when life gets hard. Different people can provide support for different things		PP: Knowing who you can count on to support your baby is this week’s personal project. Write down who those people are on worksheet 10.3		Think about who supported you this week if you were having a difficult day. Text us what kind of support they gave you	
	11	It is important to have your needs met and express what you need. Positive, clear, and direct requests are the best way to communicate. Practice being assertive		PP: This week try to ask for something from anyone—even your home visitor! What way of asking will help get your needs met—passive, aggressive, and assertive?		Reply and let us know your mood for today on a scale of 1 to 9 (with 9 being the best)	
**Planning for the future**							
	12	Creating a healthy reality for you and your baby must do with your activities, your thoughts, and your contact with others		Congratulations on finishing the MB! On a scale of 1 to 9, how helpful were texts in reminding you about main ideas and personal projects (9 is most helpful)?		Congratulations on finishing the MB! On a scale of 1 to 9, how helpful were texts in reminding you about main ideas and personal projects (9 is most helpful)?	

^a^HV: home visiting.

^b^PP: personal project.

### Measures

Demographic information included age, race or ethnicity, language spoken at home, country of birth and years in the United States, marital status, education, employment, and annual income. Pregnancy history assessed for pregnancy planning, pregnancy length, and previous childbirth.

### Feasibility of MB-TXT

MB-TXT feasibility was defined by HV adherence to the MB-TXT protocol, which included postsession documentation of the participant session data (ie, date when the MB 1-on-1 session was conducted), whether the personal project for each MB 1-on-1 session was completed [yes/no], text message ratings for usefulness and understanding, and optional notes), and the triggering of SMS text messages within 7 days of completing each in-person MB 1-on-1 session.

All self-monitoring SMS text messages invited a response from the participants and were used as a proxy measure of engagement and readability of the SMS text messages. Six of the 12 self-monitoring SMS text messages invited participants to provide a numerical rating of their stress (session 1; 9=most stressed to 1=least stressed), their mood (sessions 2, 5, 8, and 11; 9=best day ever to 1=worst day ever), or overall helpfulness of the SMS text messages (session 12; 9=most helpful to 1=least helpful). The remaining 6 self-monitoring SMS text messages invited participants to respond with a message describing how they applied the intervention skills taught during the MB 1-on-1 sessions.

### Acceptability of MB-TXT

Participants’ perceived utility and comprehension of the SMS text messages was measured using a 4-point Likert scale that assessed the usefulness (ie, 4=very useful to 1=not at all useful) and understanding (ie, 4=totally understood to 1=did not understand at all) of each SMS text message. Participant engagement in the MB-TXT was based on the number of SMS text messages sent by the participant in response to the self-monitoring texts that prompted a response (eg, *Are you working on creating your list of pleasant activities you like to do? Reply Y or N*).

### SMS Platform

HealthySMS [39] is a web-based platform designed to send health-related SMS text messages. The platform includes a dashboard that allows administrators to set up users as individual recipients or within groups, to trigger (ie, initiate) texts, to view planned activities (eg, upcoming messages) or generate data (eg, messages received by users), and to track project-specific information, such as home visitor entries of MB 1-on-1 session data. Home visitors received formal training on the MB 1-on-1 intervention before completing the training on the HealthySMS platform.

### Design

Participants were referred by their HV programs based on the study eligibility criteria, which included (1) being pregnant, (2) English or Spanish speaking, (3) >16 years, (4) and the ability to receive SMS text messages. Home visitors in each HV program identified and referred clients to the research team; research team members followed up with participants by telephone to confirm eligibility and to obtain informed consent. Before any study activities, informed consent was obtained from all participants, including clients and home visitors. Informed consent was obtained via Research Electronic Data Capture (REDCap) [40,41] for web-based consent, and verbal consent was obtained over the phone (yes or I agree) and documented by a research team member. All participants were mailed a copy of their consent form. A waiver of consent for parents or guardians of participants under 18 years of age was granted by the Northwestern University Institutional Review Board. In the US state where the study was conducted, parent or guardian consent was not required for a client under 18 years to enroll in a HV program. Participants had the option to opt out of the study activities by notifying the study team at any time.

After completing each of the MB 1-on-1 sessions, home visitors were instructed to log-in to the HealthySMS platform to enter session data and trigger session-specific SMS text messages. Once triggered, the asynchronous SMS text messages for each MB 1-on-1 session were automatically sent in a linear and sequential order, at different times of the day within a 12-hour block of time each day, 36-72 hours apart.

## Results

### Feasibility

Feasibility was defined by home visitors’ adherence to the MB-TXT protocol, which instructed them to enter session data into the HealthySMS platform within 7 days of each of the MB 1-on-1 sessions. MB-TXT SMS text messages were triggered, on average, 26.18 (SD 14.40) days following the MB 1-on-1 session (range 5.50-61.17 days).

### Response Rates

Overall, 68% (19/28) of participants completed the MB 1-on-1 intervention and received up to 36 MB-TXT SMS text messages. Of the 32% (9/28) remaining participants, 28% (8/28) did not complete the MB 1-on-1 intervention (ie, received fewer than 36 MB-TXT SMS text messages owing to dropout or premature termination). The self-monitoring SMS text messages were the only SMS text message type used to invite a participant response (Table 3). Almost all participants (25/28, 89%) responded to at least one of the 12 self-monitoring MB-TXT SMS text messages, with all participants in the sample responding to an average of 8.52 (SD 6.35) text messages. Of the 11% (3/28) of participants who did not respond to any of the self-monitoring SMS text messages, 7% (2/28) were foreign-born and 67% (19/28) were Spanish-speakers. Of note, however, these 7% (2/28) of participants did not complete the MB 1-on-1 intervention. No other group differences emerged between those who did and did not respond to the self-monitoring SMS text messages.

**Table 3:** Participant responses to Mothers and Babies Plus Text Messaging stress and mood rating text messages (N=28).

Session	Text message	Response rate, n (%)	Mean (SD)	Range	Narrative responses
1	How stressed do you feel when you think about becoming or being a mom? Reply 9 to 1 (with 9 being the most stressed and 1 the least stressed)?	16 (57)	3.64 (2.44)	1-8	“Because I have to work out whether or not I’m going to go back too [sic] work after giving birth or sit it out for a few months...and putting the new babysitting plan in action if I decide to go back to work; It feels good. I love being a mom, my kids, my life”
2	Reply and let us know your mood for today on a scale of 1-9 (with 9 being the best day ever, 5 being average, and 1 worst day ever).	18 (66)	7.06 (1.43)	4-9	“Today my son graduated from kindergarten. I’m so proud and excited!”
5	Reply and let us know your mood for today on a scale of 1-9 (with 9 being the best).	17 (61)	7.5 (1.09)	6-9	“I feel good and am present”
8	Reply and let us know your mood for today on a scale of 1-9 (with 9 being the best).	19 (68)	7.59 (1.77)	2-9	“Just got back from ______ [and the] train was 2 hours late—they lost my luggage”
11	Reply and let us know your mood for today on a scale of 1-9 (with 9 being the best).	15 (55)	7.18 (2.14)	2-9	N/A^a^

^a^N/A: not applicable.

Consistent with the MB 1-on-1 content, the self-monitoring SMS texts invited participants to respond with a numerical rating of stress (session 1; 9=most stressed to 1=least stressed) and mood (sessions 2, 5, 8, and 11; 9=best day ever to 1=worst day ever). All participants received the session 1 self-monitoring text message, with 57% (16/28) responding to these SMS text messages with an average stress rating of 3.64 (SD 2.44). MB-TXT mood ratings (for MB 1-on-1 sessions 2, 5, 8, and 11) were high, ranging from 7.06 to 7.59 out of 9. Session 12 self-monitoring SMS text message assessed the overall helpfulness of the MB-TXT, with respondents reporting high ratings (mean 8.22, SD 1.30; range 5-9). In 3 instances participants provided a narrative response instead of a numerical rating (eg, *Yes it was* in response to session 12 helpfulness of the MB-TXT) or as context to their numerical rating (eg, “...3, because I have to work out whether or not I’m going to back too [sic] work after giving birth or sit it out for a few months and putting the new babysitting plan in action if I decide to go back to work” in response to the session 1 inquiry about stress related to motherhood).

Response rates for the self-monitoring SMS text messages that invited a response on how participants were implementing intervention skills ranged from as low as 20% to as high as 65%. In their responses, participants provided examples of the pleasant activities (eg, “Yes, I crocheted on my son’s blanket while we listened to music”), positive thoughts (eg, “I must take care of myself so I can take care of those I love”), and type of contacts (eg, “Contact with my newborn. My baby was born”) they engaged in (Table 4). Skill reinforcement and homework reminder SMS text message types did not invite a participant response; however, 21 and 18 responses describing participant reactions or reflections to the texts, respectively, were received.

**Table 4:** Participant responses to Mothers and Babies Plus Text Messaging self-monitoring text messages.

Session	Topic	Text message	Narrative responses
4	Pleasant activities What do you like to do? Overcoming obstacles	Were you able to schedule or complete a pleasant activity? Reply Y/N (If Y, also text us what you did!)	“Yes, dancing.” “Yes, I went shopping for Christmas gifts (Spanish)” “Yes, I crocheted on my son’s blanket while we listened to music” “Yes, I was able to take my kids to the park and teach my daughter how to rollerblade and jump rope” “Yes, I went shopping and to a family BBQ”
6	Thoughts What are thoughts? Helpful/positive thoughts	Text us a positive thought you have about becoming or being a mom	“I am excited to see my baby’s eyes and smile” “I must take care of myself so I can take care of those I love” “I’m doing a good job! Motherhood is challenging and it [is] ok to struggle!!!” “My son is cute” “I’m looking forward to being an even better mom than before. Watching and growing with my daughter. Being there for both my children. Things are looking up as I become more positive about becoming a parent. I’m even thinking of a career change as a result”
9	Contact with others Breaking the cycle between negative mood and fewer positive contacts	Tell us about one positive contact you had this week. Text us who you were with and what you did together?	“On Monday my dad and my brother came to my house we had dinner together and we talk about our childhood” “I had positive contact with my children. We played with blocks, I read stories and we had a great day” “My friend Mara met me at the park and walked back home with me. She walked my dog while I pushed the stroller” “Contact with my newborn. My baby was born”

### Acceptability

A total of 293 MB-TXT sets of 3 messages were triggered upon completion of the MB 1-on-1 session. A total of 879 MB-TXT messages were sent through the HealthySMS platform to all 28 participants (mean 31.39, SD 7.98; range 9-36). Overall, 68% (19/28) of participants were sent the full dose of MB-TXT text messages (ie, 684 SMS text messages), given that they attended all 12 MB 1-on-1 sessions. The remaining 32% (9/28) of participants who did not complete all 12 MB 1-on-1 sessions received fewer than 36 MB-TXT SMS text messages, given that they were designed to be triggered postsession. Acceptability assessments indicated that SMS text messages were rated as useful to very useful (range 64%-86.3%) and understandable to totally understandable (range 80%-100%).

## Discussion

### Principal Findings

The MB was recently recognized by the USPSTF [12] as an evidence-based prevention of PPD intervention that should be recommended for high-risk perinatal women. This report contributes to a growing line of research focused on MB adaptations to meet the needs of perinatal women and the agencies that serve them. This study examined the acceptability and feasibility of the MB 1-on-1 intervention delivered to HV clients with added SMS text messages that reinforced skills taught in each in-person session, reminded participants of between-session homework, and focused on self-monitoring of symptoms and the application of skills to their daily lives. In addition, self-monitoring SMS text messages, which invited participants to respond, served as a proxy measure of engagement with the MB-TXT program. As expected, participants found the 36 MB-TXT SMS text messages to be highly acceptable and engaged with the program by responding to more SMS text messages than required. In fact, most participants responded to at least one text message, and text messages were rated as both useful and easily understandable, which is critical, especially when aiming to reinforce skills beyond the therapeutic session and attempting to keep the users engaged with the digital approach. Although the high acceptability of the MB-TXT was consistent with previous reports using SMS interventions with perinatal populations, participants in this study engaged at higher rates with the MB-TXT. By comparison, Broom et al [25] reported that 45% of postpartum participants responded to at least one of the treatment adjunct text messages (vs 25/28, 89% of participants in this study) and only 7.30% (vs 55%-68%; 15-19 participants in this study) responded to SMS text messages that allowed responses.

Two key components of the MB prevention intervention are: (1) teaching women how to become aware of their emotional states and related behaviors and (2) practice skills taught in each session with the goal of reducing their risk of PPD. These principles were targeted with MB-TXT self-monitoring and personal project reminder text messages. On the basis of the participants’ engagement with the MB-TXT, about 50% of the women affirmed that they were able to implement the skills taught through MB 1-on-1, a finding that is consistent with previous reports that used SMS to enhance behavioral interventions [36,42]. Similarly, home visitors indicated that approximately 50% of the women completed session-specific personal projects, which exceeds the rates found in previous MB trials [43].

The success of MB-TXT was largely dependent on the home visitors’ engagement with the HealthySMS platform. In this study, the home visitors’ level of adherence to accessing the platform within 7 days of the MB 1-on-1 session served as a feasibility measurement. Although most of the home visitors accessed the HealthySMS platform and the participants, as a result, received the full allotted set of MB-TXT SMS text messages, fewer than expected were able to access it within the protocol’s timeframe of 7 days. These implementation difficulties were often owing to logistical (eg, time efficiency by *saving* all data entry for a nonfield day), technological (eg, failure to understand how to use the HealthySMS platform), and personal barriers (eg, forgetting to trigger messages within 7 days). Regardless, these noted barriers suggest that home visitors may need additional resources to support their role in MB-TXT implementation. For instance, there were 6 participants for whom research staff needed to regularly assist their home visitors by entering session data into the HealthySMS platform. Four of these participants dropped out of their respective HV programs and ceased to receive the full dose of the MB-TXT. There were also participant and systemic events that likely contributed to challenges in feasibility (eg, disconnected participant telephone numbers or the blocking of the MB-TXT phone number and loss of funding by home visitation agencies and home visitors no longer being affiliated with their agency, respectively).

### Limitations

In addition to the noted challenges of implementing the MB-TXT as an enhancement to the MB 1-on-1 intervention, there are several limitations to consider. The factors that contribute to acceptability ratings remain unknown. That is, the valence of these ratings may be due to external factors not associated with MB-TXT, such as home visitors’ understanding of and competency in delivering the MB 1-on-1 or participants’ desire to respond favorably to maintain their relationship with the home visitor (ie, social desirability). Related, acceptability ratings were gathered in aggregate by session number and not by text message type. As such, it is unclear whether a specific text message or message type may have been rated differently (eg, for a specific session, the skill reinforcement text may have been experienced as very useful, but the self-monitoring SMS text message may not have been). This lost data point has implications for future iterations of the MB-TXT and the research team’s goal of improving the program content and impact on PPD. The wording of SMS text messages may have also influenced how participants engaged in MB-TXT. For instance, the skills reinforcement self-monitoring messages that invited participants to share how they applied intervention skills to their daily lives may have inadvertently discouraged those who did not implement the skills from submitting a response. As such, it is difficult to ascertain whether those who did not respond were participants who, in fact, did implement the skills but failed to respond versus those who may have struggled to implement the skills and did not want to provide that feedback or who simply ignored the invitation to provide a response.

### Conclusions

The findings of this study are promising as low-cost evidence-based digital interventions are being developed with the goal of reaching underserved communities of perinatal women at risk for PPD. Future iterations of MB-TXT will aim to integrate lessons learned from this study, especially as they relate to digital interventions that rely on or integrate human support as part of implementation.

## Abbreviations

eMB: Mothers and Babies Web-based Course
HV: home visiting
MB: Mothers and Babies
MB-TXT: Mothers and Babies Plus Text Messaging
PPD: postpartum depression
REDCap: Research Electronic Data Capture
USPSTF: United States Preventive Services Task Force
